# Beneficial Effects of Salt on Halophyte Growth: Morphology, Cells, and Genes

**DOI:** 10.1515/biol-2019-0021

**Published:** 2019-06-24

**Authors:** Fang Yuan, Yanyu Xu, Bingying Leng, Baoshan Wang

**Affiliations:** 1 Shandong Provincial Key Laboratory of Plant Stress, College of Life Sciences, Shandong Normal University, Ji’nan, Shandong, 250014, P.R. China

**Keywords:** Hydrophobic barriers, Ion compartmentalization, Molecular, Salt gland, Salt tolerance

## Abstract

Halophytes can survive and complete their life cycle in the presence of ≥200 mM NaCl. These remarkable plants have developed various strategies to tolerate salinity and thrive in high-salt environments. At the appropriate levels, salt has a beneficial effect on the vegetative growth of halophytes but inhibits the growth of non-halophytes. In recent years, many studies have focused on elucidating the salt-tolerance mechanisms of halophytes at the molecular, physiological, and individual level. In this review, we focus on the mechanisms, from the macroscopic to the molecular, underlying the successful growth of halophytes in saline environments to explain why salt has beneficial effects on halophytes but harmful effects on non-halophytes. These mechanisms include the specialized organs of halophytes (for example, ion compartmentalization in succulent leaves), their unique structures (salt glands and hydrophobic barriers in roots), and their salt-tolerance genes. We hope to shed light on the use of halophytes for engineering salt-tolerant crops, soil conservation, and the protection of freshwater resources in the near future.

## 1 Introduction

Salt water accounts for approximately 97% of the Earth’s water supply, and humans can only use 1% of the fresh water found worldwide [1]. Saving fresh water and making good use of salt water pose new challenges, especially in arid and semi-arid countries. Halophytes naturally grow in saline environments [2,3], and some species, such as mangroves, can even grow in seawater [4]. Halophyte plants can be used as forage grasses, in medicines, as vegetables, and as papermaking materials [1]. Therefore, investigating the mechanisms by which halophytes tolerate saline environments is crucial for sustainable development.

Salt can damage plants through its osmotic effect (physiological drought under high-salinity conditions), ion toxicity (especially Na^+^ and Cl^-^), and secondary stresses such as oxidative stress [5,6]. Halophytes and non-halophytes show distinct differences in maximum salt tolerance [7, 8, 9]. Plants that can survive and complete their life cycle in a salt concentration of ≥200 mM NaCl are usually defined as halophytes [5,10,11, 12, 13]. Halophytes actively control the uptake, storage, exclusion, and secretion of ions under saline conditions [14, 15, 16, 17]. The most salt-tolerant halophytes such as *Suaeda salsa* can complete their life cycle in soils containing 200 to 500 mM NaCl [18, 19, 20], whereas non-halophytes show limited salt tolerance and can be damaged in soils with NaCl concentrations <50 mM [21].

Halophytes do not simply tolerate high-salt conditions. True halophytes thrive at the appropriate salt concentrations and show optimal growth in the presence of significant amounts of NaCl, *e.g*, 200 mM for *S. salsa* [22,23], 150 mM for *Chenopodium quinoa* [24], and 100 mM for *Cakile maritima* [25]. The halophyte *Plantago crassifolia* exhibits highly efficient responses to salt stress during early seedling development [26,27]. Appropriate salt concentrations can promote the vegetative growth of halophytes and are conducive to the completion of their life cycle, as described by Flowers & Colmer [10]. Salt has a beneficial effect on halophytes, as they grow larger and more rapidly in the presence of the appropriate salt concentration, compared to conditions with little or no salt [20]. More specifically, the majority of halophytes benefit from the presence of high concentrations of salt during processes ranging from seed germination to seedling growth. For example, many halophytes such as *Cakile maritima* [25] and *Chloris virgata* [28] have been shown to have higher germination percentages at slightly elevated salinity levels (0.5% NaCl, or around 50–90 mM) vs. distilled water [28,29]. In addition to seed germination, appropriate NaCl concentrations also enhance the seedling growth of halophytes compared to non-salt conditions. This is evidenced by higher seedling biomass, larger leaf area [30], and enhanced photosynthetic efficiency [17,31] and yield, thus leading to increased seed production for the next generation [18,19].

By contrast, non-halophytes are salt sensitive and suffer from salt-induced damage. These plants are classified as salt-sensitive and salt-tolerant non-halophytes based on their level of salt tolerance. Plants in both categories show inhibited growth under saline conditions, but salt-sensitive non-halophytes, such as soybean and rice, may suffer irreparable damage in response to low concentrations of NaCl (less than 50 mM) [32,33], whereas salt-tolerant non-halophytes such as cotton, beets, and barley can tolerate higher salt concentrations (200–300 mM NaCl) [34, 35, 36]. However, all non-halophytes show decreased biomass when grown in the presence of salt with one exception: *Eutrema salsugineum* (formerly misclassified as *Thellungiella salsuginea*, Brassicaceae [37,38]). This plant is widely considered to be a model halophyte [39] because it has a certain degree of salt tolerance and was reported to survive under 250 mM NaCl conditions [40,41], although its growth sharply declines with increasing NaCl level [42,43]. Studies of *E. salsugineum* performed over the past 15 years have contributed to our understanding of salt tolerance mechanisms in halophytes.

Why does the appropriate salinity level enhance the vegetative growth of halophytes and inhibit the growth of non-halophytes? Do halophytes have special characteristics that allow them to adapt to saline environments? In the past decade, many studies have investigated possible underlying mechanism. In the current review, we focus on the vegetative growth of halophytes to illustrate the mechanisms underlying the robust growth of halophytes in saline environments, from the morphological to the cellular and molecular levels.

## 2 Morphological, cellular, and sub-cellular adaptations

**Figure 1:**
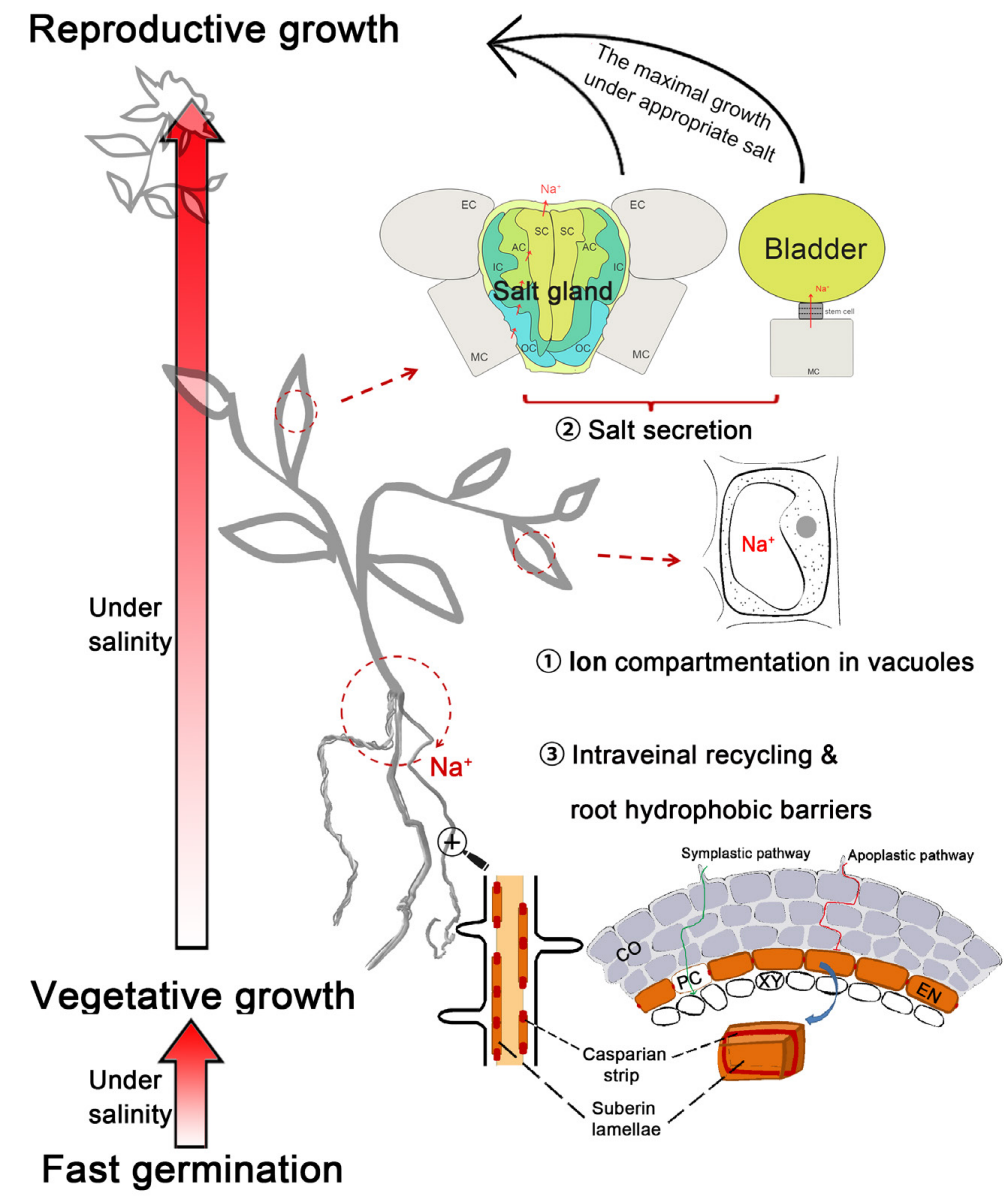
Salt-tolerance mechanisms in halophytes. Seeds that rapidly germinate under saline conditions benefit from dimorphism or mobilization. Vegetative growth is maximal and reproductive growth is stimulated under appropriate salt concentrations because of the following salt-tolerance mechanisms: 1) ion compartmentalization; 2) salt secretion; and 3) ion intraveinal recycling and the root apoplastic barrier. In the first mechanism- ion compartmentalization- Na^+^ actively accumulates in the vacuoles, thus preventing protoplast damage. The second mechanism- salt secretion- is described in Yuan *et al* [3], which showed the typical multi-cellular salt gland and salt bladder. SC, secretory cell; AC, accessory cell; IC, inner cup cell; OC, outer cup cell; MC, mesophyll cell; EC, epidermal cell. The third mechanism- the root apoplastic barrier- includes the Casparian strip and suberin lamellae, which can effectively block the apoplastic pathway. Ions can only enter endothelial cells via passage cells (PC), *i.e*, the symplastic pathway. XY, xylem; CO, cortex; EN, endodermis; PC: passage cell. The plant was drawn with Photoshop CS6.

All plants, including non-halophytes, compartmentalize excess ions into their vacuoles, which is considered the physiological foundation of salt tolerance in all plants [44]. Halophytes have evolved several specific structures or mechanisms to adapt to saline environments (Fig. 1). However, non-halophytes have not evolved the unique morphological features needed to cope with salt stress, and if forced to live in saline soil, their biomass is reduced and they cannot complete their life cycles. By contrast, halophytes can survive high-salt conditions due to leaf succulence and the functions of specialized organs (*e.g*, salt glands, as described below). There are three types of halophytes: euhalophytes, recretohalophytes, and pseudohalophytes [45]. Euhalophytes such as *Kalidium foliatum* and *S. salsa* are salt accumulators that can take up large amounts of ions and compartmentalize them in vacuoles to maintain cell turgor. These plants also develop leaf or stem succulence when the soil water potential is low [20].

**Figure 2:**
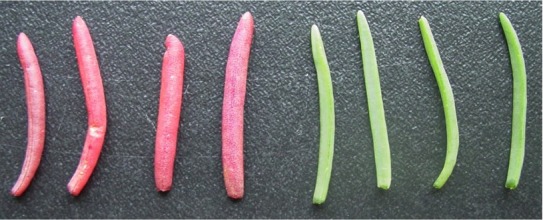
The succulent blades of euhalophyte *Suaeda salsa* grown in intertidal zone (left, red leaves) and inland saline soils (right, green leaves) of the Yellow River Delta (N 37°25′; E 118°54′).

Leaf succulence is a typical visible characteristic of euhalophytes such as *S. salsa* under high-salinity conditions (Fig. 2) [46,47], although this feature is not unique to halophytic plants as certain xerophytes, such as cacti and *Kalanchoe daigremontiana* also have succulent leaves under drought conditions. However, halophytes and xerophytes have evolved different strategies leading to the formation of succulence. Under saline conditions, ion accumulation in vacuoles results in succulence, which may be caused by the presence of carbon as a driving force and ion compartmentalization to relieve salt damage. For example, *S. salsa* actively accumulates ions and proline in its vacuoles and cytosol to reduce plant water potential [48]. A possible mechanism underlying leaf succulence in *S. salsa* is suggested by the finding that the presence of aquaporins in the plasma membrane is correlated with Na^+^ accumulation in the vacuole [23,49,50]. Under drought conditions, however, succulence is induced by the accumulation of organic compounds such as malate via a carbon gradient [51].

Halophytes and non-halophytes show distinct differences in ion compartmentalization. Photosynthesis and chloroplasts in non-halophytes are markedly damaged by salinity due to a weak ion compartmentalization [52]. For example, in *Arabidopsis thaliana*, electron transport though photosystem II is dramatically inhibited and nonphotochemical quenching of chlorophyll fluorescence increases in response to 150 mM NaCl [39]. By contrast,

the chloroplasts and mitochondria of halophytes are protected under salt-stress conditions due to a strong ion compartmentalization. The ultrastructure of thylakoids in the chloroplasts of two euhalophytic species *Haloxylon ammodendron* and *Suaeda physophora* showed no observable damage when treated with 700 mM NaCl [17]. In the halophyte *Artemisia anethifolia*, the thylakoids in chloroplasts were intact, and the number of cristae in the mitochondria did not decrease until the plants were treated with 200 mM NaCl [53]. The halophyte *Suaeda altissima* also retained normal chloroplast function under 750 mM NaCl conditions [31].

The two other types of halophytes are considered to be salt regulators. Recretohalophytes can secrete excessive ions via specific salt-secreting structures, *e.g*, salt bladders in *Atriplex centralasiatica* [54] and salt glands in *L. bicolor* [55,56]. These unique epidermal structures distinguish these plants from other halophytes and all non-halophytes [3,57]. Vesicle transport is the main pathway for salt secretion [2,58]. The subcellular structures of recretohalophytes also exhibit specific characteristics. Most reports have focused on salt-secretory ultrastructures such as highly developed plasmodesmata, mitochondria, vesicles, the lack of chloroplasts, cuticles, and so on (this information can be found in Yuan *et al* [3] and Shabala *et al* [54]). A review by Dassanayake [59] discusses the morphology and evolution of salt glands, suggesting that these structures emerged independently at least 12 times in recretohalophytes.

The roots of non-halophytes and halophytes behave quite differently. In root cells of the non-halophyte common bean (*Phaseolus vulgaris*), the addition of 80 mM NaCl leads to membrane vesiculation and increased solute leakage [60]. By contrast, the roots of pseudohalophytes such as mangroves in the Rhizophoraceae family and reeds in the *Gramineae* family show high salt exclusion ability, thereby protecting the shoots from salinity. The possible mechanism underlying salt exclusion in plants such as reeds is described as interveinal recycling and apoplastic barriers in the roots. During interveinal recycling, the Na^+^ absorbed by roots is transported into the shoots through xylem vessels and is then loaded into the phloem by HKT1 (a high-affinity K^+^ transporter) [61,62]. Finally, this Na^+^ is unloaded back into the soil by SOS1 (a plasma membrane Na^+^/H^+^ antiporter) [63,64] in roots cells [65,66] (detailed in the “Salt-tolerance genes” section). In recent years, lignin and suberin lamellae in the root endodermis have also been shown to be involved in the salt exclusion pathway. Root hydrophobic barriers play an important role in salt exclusion in *Avicennia officinalis* [4]. The same group reported that, although rice is a representative non-halophyte, it can also tolerate low concentrations of salt (50–100 mM NaCl), mainly due to the presence of apoplastic transport barriers in the roots [67].

As mentioned in the Introduction, *E. salsugineum* is a special type of halophyte that has been used as a model plant to unravel the molecular mechanisms of salt tolerance in halophytes [68,69]. Although this plant does not possess the typical characteristics of halophytes (such as salt glands or salt bladders) and shows a marked decrease in vegetative growth under high salinity, studies of *E. salsugineum* have shed light on the mechanisms underlying salt tolerance. Under high-salt conditions, *E. salsugineum* undergoes differential regulation of Na^+^/K^+^ ions and re-establishes Na^+^/K^+^ homeostasis [70], including a reduction in Na^+^ absorption [71] and an increase in Na^+^ compartmentalization [72,73]. The genes controlling Na^+^ absorption are described in the following section and listed in Table 1. The osmotic balance in *E. salsugineum* can also be maintained by proline accumulation in addition to ion accumulation [74], which helps this plant survive in saline environments.

**Table 1:** Genes involved in Na^+^ influx and the three salt tolerance mechanisms of halophytes

Salt-tolerance mechanism	Gene	Likely function in salt tolerance	Halophyte species	References
Na^+^ influx	*HKT1*	High-affinity K^+^ transporter 1	*Suaeda salsa*	[92]
			*Salicornia dolichostachya*	[111]
			*Leptochloa fusca*	[112]
			*Aeluropus lagopoides*	[113]
	*AKT1*	Inward-rectifying K channel 1	*Suaeda maritima*	[114]
	*KUP*/*HAK*/*KT*	KUP/HAK/KT type transporter	*Suaeda maritime*	[115]
			*Eutrema salsugineum*	[71]
	*KT*	Potassium transporter	*Reaumuria trigyna*	[104]
			*Limonium bicolor*	[56]

1) Ion compart-	*NHX*	Encodes a vacuolar-type Na^+^/H^+^ antiporter that	*Limonium gmelinii*	[116]
mentation in vacuoles		is located on the vacuolar membrane and pumps	*Karelinia caspica*	[81]
		excessive Na^+^ into the vacuole to avoid toxic Na^+^	*Salicornia brachiate*	[114]
		concentrations in the cytoplasm.	*Aeluropus littoralis*	[118,91]
	*CLC*	Chloride channel on vacuolar membrane	*Mesembryanthemum crystallinum*	[119]
	*AQP*	Encodes aquaporin	*Sesuvium portulacastrum*	[76]
			*Suaeda salsa*	[50]

2) Salt secretion	*SOS1*	Encodes a Na^+^/H^+^ antiporter located on the plasma membrane that pumps excess Na^+^ out of the cell.	*Avicennia marina*	[120]
	*HA1*	PM H^+^-ATPase	*Avicennia marina*	[120]
	*NHX*	Na^+^/H^+^ antiporter on the vacuolar membrane	*Avicennia marina*	[120]
	*VAMP*	Vesicle-associated membrane protein	*Limonium bicolor*	[56]
	*CLC*	Chloride channel on the plasma membrane	*Limonium bicolor*	[56]
	*PIP* and *TIP*	Aquaporin genes	*Avicennia officinalis*	[121]

3) Intravein recycling and root hydrophobic barriers	*SOS1*	Encodes a Na^+^/H^+^ antiporter located on the plasma membrane that plays a role in Na^+^ efflux from roots	*Salicornia dolichostachya*	[111]
	*AoCYP86B1*	Encodes cytochrome P450 that regulates suberin biosynthesis and prevents some Na^+^ from entering the roots	*Avicennia officinalis*	[4]

In short, halophytes have evolved several structural or ultrastructural adaptations to salt stress, whereas non-halophytes do not develop these adaptive structures, and their ultrastructure is significantly injured under low-salt conditions. Therefore, specific cellular and subcellular structures facilitate the strong growth of halophytes under the appropriate salt concentrations.

## 3 Salt-tolerance genes

All traits, including salt tolerance and salt sensitivity, are ultimately controlled by genes. Certain salt tolerance genes are constitutively expressed in halophytes while other genes are induced by salt [75], exhibiting increased expression under salt treatment [76,77]. Although many reports involving salt-tolerance genes have focused on non-halophytes such as *Arabidopsis* [64,78,79] and rice [80], we will concentrate on salt-tolerance genes in halophytes. Table 1 lists the genes involved in Na^+^ transport across the membrane and the three salt-tolerance mechanisms used by halophytes (also see Fig. 1). Na^+^ flux occurs from root to leaf in halophytes based on the genes described to date. Na^+^ may enter the cell by HKT1, KT, KUP/HAK/KT-type transporters, AKT1-type channels, and NSCCs (nonselective cation channels). To avoid salt damage to the cytoplasm, many genes involved in the three salt-tolerance mechanisms are upregulated, such as *NHX* (encoding a vacuolar-type Na^+^/H^+^ antiporter that participates in ion compartmentation in vacuoles);

SOS pathway genes such as *SOS1*; *PIP* (aquaporin involved in salt secretion); and *cytochrome P450* (involved in the root hydrophobic barrier).

To date, to the best of our knowledge, only one halophyte gene has been tested in a halophyte to verify its function. Silencing *KcNHX1* in the halophyte *Karelinia caspica* led to reduced tolerance to high concentrations of NaCl, suggesting that KcNHX1 plays an essential role in the response of *K. caspica* to salt stress [81]. Most of the same genes may be present in halophytes and non-halophytes but exhibit different expression patterns due to different long-term survival strategies [82]. Therefore, all salt-tolerance genes that have been cloned in halophytes to date have been tested by heterologous expression in non-halophytes to explore their functions [83, 84, 85, 86, 87, 88]. The highest concentration of NaCl that these transgenic plants could tolerate was reported as 400 mM [89,90]. For example, transgenic tobacco (*Nicotiana tabacum*) transformed with *AlNHX* (encoding a vacuolar-typed Na^+^/H^+^ antiporter) from the halophyte *Aeluropus littoralis* exhibited high salt tolerance (400 mM NaCl) [91]. Transgenic tobacco also compartmentalized more Na^+^ in its roots than wild type tobacco to maintain a relatively high K^+^/Na^+^ ratio in its leaves [91]. Overexpression of a similar gene *SsNHX1* (encoding a putative vacuolar Na^+^/H^+^ antiporter) from *Salsola soda* allowed *Medicago sativa* to survive in high concentrations of NaCl (up to 400 mM) due to improved Na^+^ sequestration in the vacuole [90].

In addition to the role of NHX genes in ion compartmentation, studies in non-halophytes have also verified the functions of many other groups of halophyte genes controlling primary salt-tolerance traits, showing that heterologous expression of these genes significantly improved the salt tolerance of these plants. The first group of genes includes *HKT1* (encoding a high-affinity K^+^ transporter) and *SOS1* (encoding a plasma membrane Na^+^/ H^+^ antiporter). Transgenic *Arabidopsis* transformed with *SsHKT1;1* from *S. salsa* showed enhanced salt tolerance and increased K^+^ concentrations in shoots [92]. Transgenic tobacco harboring *SbSOS1* from *Salicornia brachiata* showed a high degree of salt tolerance, growing in 200 mM NaCl [93].

The second group of genes, including H^+^-pyrophosphatase and vacuolar ATPase genes, is involved in energy supply. For example, transgenic *Arabidopsis* transformed with *SsVP* (encoding a vacuolar H^+^ -pyrophosphatase) from *S. salsa* [94] or *KfVP1* (encoding H^+^-pyrophosphatase) from *Kalidium foliatum* [95] showed increased salt tolerance due to enhanced V-ATPase and V-PPase activity. Transgenic rice transformed with *SaVHAc1* (a vacuolar H^+^-ATPase subunit c1 gene) from the halophyte *Spartina alterniflora* performs better under salt stress than control [96].

The third group of genes is involved in the ROS scavenging system. Transgenic tobacco transformed with *SbpAPX* (encoding Peroxisomal Ascorbate Peroxidase) from *S. brachiata* showed enhanced vegetative growth compared to wild type when grown at 300 mM NaCl [97]. Transformation with *Ss.sAPX* (encoding a stromal ascorbate peroxidase) from *S. salsa* improved the growth of Arabidopsis plants under high-salt conditions [84].

The remaining groups of genes are related to plant hormones and aquaporin. Transgenic tobacco expressing high levels of *SbASR-1* (encoding abscisic acid stress ripening-1) from *S. brachiata* showed better germination and seedling growth than wild type when grown on 400 mM NaCl [89]. Transgenic tobacco harboring *SpAQP1* (aquaporin-related gene induced by salt) from *Sesuvium portulacastrum* showed enhanced seed germination and root growth under high-salt conditions due to increased antioxidant enzyme activity [76].

The heterologous expression of halophytic salt-tolerance genes improves salt resistance in non-halophytes to some degree, but transgenic plants often cannot finish their life cycles in naturally saline soils due to the great spatial and temporal variation of salt content. Moreover, to the best of our knowledge, no transgenic non-halophytes show typical halophyte characteristics such as improved growth under the appropriate salt concentration. In general, salt-tolerance traits are controlled by a series of genes rather than one or two genes. Therefore, it might be necessary to identify salt-tolerance gene networks and explore their effects under controlled conditions.

## 4 Conclusions and Perspective

The vegetative growth of halophytes can benefit from appropriate salt concentrations. Although different halophytes have evolved diverse salt-tolerance mechanisms, these can primarily be divided into three categories: the use of specialized organs (succulent leaves via ion compartmentalization), unique structures (salt glands and hydrophobic barriers in roots), and salt-tolerance genes. In this review, we focused on the mechanisms that could explain the beneficial effects of salt on vegetative growth in halophytes (*i.e*, better and more rapid growth than under non-salt conditions, resulting in increased seed production), including the morphological, cellular, and molecular aspects of these mechanisms. Additional reviews about various salt-tolerance mechanisms can be found in [82,98,99,100]. Many reports emphasize the important role of halophytes in improving saline soil conditions and the cultivation of salt-tolerant crops [1,3,20,44,82,100,101]. Several researchers have proposed a series of possible ways to realize these dreams, such as transforming non-halophytes with salt-tolerance genes to improve their salt resistance [82]. Indeed, salt-tolerance genes isolated from halophytes are often used to transform non-halophytes.

However, it is still difficult to apply these solutions to plants grown in the field and these solutions face many challenges. To date, no glycophytes/non-halophytes transformed with salt-tolerance genes have been successfully grown in natural saline environments. On the one hand, all known salt-tolerance genes have been heterologously overexpressed in non-halophytes to clarify their functions, which is not a very precise method. The functions of salt-tolerance genes should be verified in the halophyte itself via silencing or knockout, but this type of experiment has only been reported for the halophyte *K. caspica* [81]. On the other hand, salt tolerance in halophytes is a complex trait that is controlled by gene families or networks. Transforming one or several related genes into glycophytes may not cause radical changes in salt tolerance; instead, the transformed genes must function coordinately. Nevertheless, these solutions appear feasible, but additional time is needed to carry out such experiments.

For the discovery of salt-tolerance genes and networks, high-throughput RNA-seq has been used in several halophytes such as *L. bicolor* [13], *M. crystallinum* [102,103], and *Reaumuria trigyna* [104]. Although many salt-tolerance genes have been identified in halophytes, which genes should we focus on first? Perhaps we can focus on the genes controlling primary salt-tolerance traits as mentioned in this review (such as succulent leaves, salt glands, and root hydrophobic barriers), followed by regulatory genes (such as transcription factor genes) that control these traits (e.g., Table 1) by transforming the halophyte itself. Using this procedure, we can target the key traits directly involved in salt tolerance and the corresponding phenotypes, allowing a single trait to be improved in non-halophytes via the transformation of these genes. Good transformation systems are clearly needed for this strategy and, therefore, there is an urgent need to establish such systems for use in various halophytes, such as *Leymus chinensis* and *L. bicolor* [105,106]. Based on this system, CRISPR/Cas9-mediated genome editing will likely prove to be a useful tool for verifying target gene function [107]. In addition, many recent studies have found that long non-coding RNAs play an important role in salt tolerance in plants [108, 109, 110]. Therefore, more attention should be paid to non-coding RNAs that participate in the unique salt-tolerance strategies of halophytes via high-throughput RNA sequencing.

Overall, given that the expanding saline lands threaten human existence, there are two ways to make good use of halophytes to preserve soils and fresh water: 1) increasing the planting areas of halophytes in arid and semi-arid areas to help prevent water loss and 2) transforming non-halophytes with salt-tolerance genes to enable them to tolerate irrigation with full-strength or diluted seawater in the near future.
